# IgG1-3 MuSK Antibodies Inhibit AChR Cluster Formation, Restored by SHP2 Inhibitor, Despite Normal MuSK, DOK7, or AChR Subunit Phosphorylation

**DOI:** 10.1212/NXI.0000000000200147

**Published:** 2023-08-15

**Authors:** Michelangelo Cao, Wei-Wei Liu, Susan Maxwell, Saif Huda, Richard Webster, Amelia Evoli, David Beeson, Judith A. Cossins, Angela Vincent

**Affiliations:** From the Nuffield Department of Clinical Neurosciences (M.C., W.W.L., S.M., R.W., D.B., J.A.C., A.V.), University of Oxford; Norfolk and Norwich University Hospital (M.C.); The Walton Centre NHS Foundation Trust (S.H.), Liverpool, United Kingdom; and Department of Neuroscience (A.E.), Catholic University, Rome, Italy.

## Abstract

**Background and Objectives:**

Up to 50% of patients with myasthenia gravis (MG) without acetylcholine receptor antibodies (AChR-Abs) have antibodies to muscle-specific kinase (MuSK). Most MuSK antibodies (MuSK-Abs) are IgG4 and inhibit agrin-induced MuSK phosphorylation, leading to impaired clustering of AChRs at the developing or mature neuromuscular junction. However, IgG1-3 MuSK-Abs also exist in MuSK-MG patients, and their potential mechanisms have not been explored fully.

**Methods:**

C2C12 myotubes were exposed to MuSK-MG plasma IgG1-3 or IgG4, with or without purified agrin. MuSK, Downstream of Kinase 7 (DOK7), and βAChR were immunoprecipitated and their phosphorylation levels identified by immunoblotting. Agrin and agrin-independent AChR clusters were measured by immunofluorescence and AChR numbers by binding of ^125^I-α-bungarotoxin. Transcriptomic analysis was performed on treated myotubes.

**Results:**

IgG1-3 MuSK-Abs impaired AChR clustering without inhibiting agrin-induced MuSK phosphorylation. Moreover, the well-established pathway initiated by MuSK through DOK7, resulting in βAChR phosphorylation, was not impaired by MuSK-IgG1-3 and was agrin-independent. Nevertheless, the AChR clusters did not form, and both the number of AChR microclusters that precede full cluster formation and the myotube surface AChRs were reduced. Transcriptomic analysis did not throw light on the pathways involved. However, the SHP2 inhibitor, NSC-87877, increased the number of microclusters and led to fully formed AChR clusters.

**Discussion:**

MuSK-IgG1-3 is pathogenic but seems to act through a noncanonical pathway. Further studies should throw light on the mechanisms involved at the neuromuscular junction.

## Introduction

Myasthenia gravis (MG) with antibodies against muscle-specific kinase (MuSK-MG) is an autoimmune disease that impairs neuromuscular transmission leading to widespread weakness and fatigability of skeletal muscles.^1,2^ Efficient neuromuscular transmission depends on the high-density clustering of acetylcholine receptors (AChRs) on the postsynaptic membrane at the neuromuscular junction (NMJ). AChR clustering is induced by the release of agrin, a soluble heparan-sulfate proteoglycan from the motor nerve terminal.^3,4^ Agrin binds to low-density lipoprotein receptor-related protein 4 (LRP4) which then interacts with MuSK on the postsynaptic membrane.^5^ This triggers MuSK phosphorylation and recruitment of Downstream of Kinase 7 (DOK7) leading to a phosphorylation cascade that eventually reaches rapsyn and the AChR which cocluster (reviewed in references 6, 7).

The autoantibodies against MuSK (MuSK-Abs) are mainly of the IgG4 subclass and functionally monovalent due to “fab-arm exchange.”^8^ These antibodies block the binding of agrin-LRP4 to MuSK resulting in inhibition of the phosphorylation cascade and dispersal of AChRs, as studied using the mouse C2C12 cell line in vitro.^9,10^ Although not as dominant as MuSK-IgG4-Abs, however, MuSK-IgG1, 2, or 3 subclass antibodies exist in all patients with MuSK-MG and were also reported to interfere with AChR clustering.^10^ Moreover, divalent antibodies are pathogenic in mice immunized against purified MuSK, irrespective of mouse IgG1 antibodies (the murine equivalent of IgG4).^11^

The aim of this work was to develop further insights into the potential pathogenic role(s) of MuSK-IgG1-3-Abs (MuSK-IgG1-3) and to see whether the SHP2 phosphatase inhibitor, NSC-87877,^12^ could restore the AChR clusters as it did for IgG4 MuSK-Abs.^13^

## Methods

### MuSK-Ab Titration and IgG Subclass Purifications

Plasmapheresis material was collected, with informed consent, from 8 MuSK-Ab positive MG patients. The total and MuSK-IgG1-3 titres were measured as previously described.^13^ Five chosen plasmas were pooled for IgG subclass fractionation and incubated overnight at 4°C with an IgG4 affinity matrix (CaptureSelect IgG4, Thermo Fisher, Cheshire, UK). IgG4 was eluted from the matrix with glycine buffer (0.1 M; pH 2.3) and neutralized. The flow-through fractions containing IgG1-3 were then purified by binding to protein G Sepharose (P3296, Sigma, St. Louis, USA) and eluted with glycine buffer, neutralized, and re-exposed to the IgG4 affinity matrix to remove any residual IgG4. MuSK-Ab titres were measured in each of the purified fractions by radioimmunoassay and adjusted to 0.5 nM for all experiments.

### C2C12 Myotube Cultures and AChR Cluster Counts

C2C12 mouse myoblasts (91031101-DNA-5UG, Sigma) were differentiated into myotubes with differentiation medium (DMEM with 2% fetal calf serum/horse serum).^13^ Myotubes were then incubated with purified MuSK antibody subclasses (0.5 nM) in the presence or absence of recombinant short form agrin 1:1,000 (producing approximately 50% of maximum AChR clusters). Myotubes exposed to medium only were negative controls. AChR clusters were labeled with Alexa Fluor 594 α-bungarotoxin (1:1,000; B13422, Invitrogen) and fixed in 3% formaldehyde. Twenty fields, selected with bright field, were counted for “fully formed clusters” (≥3 μm) and, in some experiments, also for “microclusters” (≥1 μm and <3 μm) using ImageJ software. When required, NSC-87877 was added with or without agrin.^13^

### MuSK, DOK7, and βAChR Phosphorylation Analysis

Phosphorylation of MuSK, DOK7, and βAChR was assessed by precipitation of each protein and Western blotting. Myotubes were starved of fetal calf/horse serum for 3 hours and incubated at 37°C with MuSK-Ab subclasses with or without agrin (1:1,000). After the appropriate incubation time, the myotubes were lysed in cold lysis buffer (10 mM Tris-HCl, 1 mM EDTA, 100 mM, NaCl, 1% Triton X-100, 1x protease (P8340, Sigma), and phosphatase (78420, Thermo Fisher) inhibitor cocktails) and centrifuged for 10 minutes at 12,600 rpm. For pulldown of MuSK exposed to MuSK-IgG1-3 or IgG4, the supernatants were incubated overnight at 4°C with Protein G Dynabeads (10004D, Thermo Fisher) without addition of further MuSK-Abs. For pulldown from cells exposed to DMEM or control IgG, a polyclonal anti-MuSK antibody AF562 (Bio-Techne, Minneapolis) was used. Mouse DOK7 was precipitated with anti-DOK7 (A7, SC-390856, Santa Cruz Biotechnology, TX), and βAChR was first labeled with biotin-α-bungarotoxin (B1196, Invitrogen) and precipitated with Dynabeads Streptavidin T1 (65601, Thermo Fisher). The precipitated proteins were eluted into SDS sample buffer for electrophoresis and Western blotting. The blots were first probed with anti-phosphotyrosine antibody 1:1,000 (4G10, Upstate Biotechnology, MA) followed by secondary rat anti-mouse native IgG HRP-conjugated antibody (ab131368, Abcam). The nitrocellulose was stripped and reprobed for MuSK, DOK7, or βAChR expression with commercial antibodies AF562, A7, and anti-βAChR/148 (sc-65813, Santa Cruz), respectively. Band densitometry was performed using ImageJ software. MuSK, DOK7, and βAChR phosphorylation levels were normalized to the levels of their respective proteins.

### AChR Membrane Expression Assay

C2C12 myotubes were exposed overnight to agrin (1:1,000) and MuSK-Ab subclasses (0.5 nM). Myotubes were incubated for 1 hour at room temperature with ^125^I-α-bungarotoxin at 1 × 10^6^ cpm/mL per well. After washing, the cells were lysed in extraction buffer (10 mM Tris, 100 mM NaCl, 1 mM EDTA, and 1% Triton X100), and the total cpm was counted on a γ-counter.

### RNA Extraction and Sequencing Analysis

Total RNA was extracted from myotubes after exposure to the antibody preparations or NSC-87877 100 µg for 2.5 hours at 37°C. Myotubes were washed with differentiation medium, and RNA was extracted using TRIzol Reagent (15596026, Thermo Fisher); contaminating genomic DNA was removed using the TURBO DNA-free kit (AM1907, Thermo Fisher) following manufacturers' instructions. After purity check, 20 µL of each sample (at 50 ng/µL) were sent in triplicates to Novogene (UK) for transcriptomic library preparation and sequencing. RNA sequences were aligned to the mus musculus genome using STAR (Spliced Alignment to a Reference). Reads were counted using featureCounts and analyzed using edgeR (Bioconductor). Data were filtered to exclude genes with <1 count per million. RNA composition was adjusted using calcNormFactors, and the coefficient of variation and dispersion was estimated. TopTags was used to obtain log-fold change data. Bigwig files were created from BAM files for viewing in the UCSC genome browser.

### Statistical Analysis

Statistical tests were performed with GraphPad Prism 6 as indicated in the figure legends. Error bars represent the standard error of the mean of 3 independent experiments unless otherwise specified. Differences with *p* values <0.05 were considered statistically significant.

### Standard Protocol Approvals, Registrations, and Patient Consents

The study of archived patient samples, as in reference 10, was approved by the Oxfordshire Research Ethics Committee A (07 Q160X/28).

### Data Availability

The authors confirm that all data supporting the findings of this study will be made available upon reasonable request to the corresponding author.

## Results

**Figure 1:**
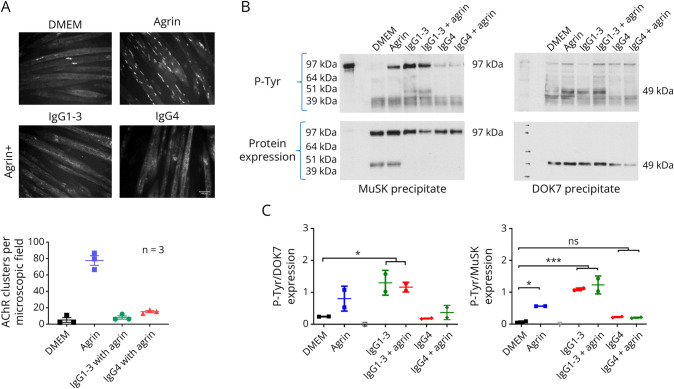
IgG1-3 and IgG4 Both Inhibit AChR Clustering but Have Opposite Effects on MuSK and DOK7 Phosphorylation (A) Myotubes were first exposed to MuSK-IgG1-3 and IgG4 subclasses followed 50 mins later by agrin. Agrin alone stimulated AChR clusters, but both IgG fractions inhibited the number of AChR clusters to the levels similar to those in DMEM alone. Scale bar = 50 μm. (B) To explore phosphorylation of MuSK and DOK7 in parallel, myotubes were exposed to MuSK-Abs IgG1-3 for 40 minutes, either with or without agrin. Representative Western blots of immunoprecipitated MuSK showed a strong phosphorylation band for both MuSK (left) and DOK7 (right) irrespective of the presence of agrin, with only faint signal for the IgG4-treated cells. The densities of the phosphorylated MuSK or DOK7 (upper gels) were normalized to those of the MuSK or DOK7 proteins after stripping and reprobing the blots (lower gels). (C) The results pooled from 2 independent experiments; MuSK-IgG1-3 increased both MuSK and DOK7 phosphorylation. One-way ANOVA with multiple comparisons against DMEM. Mean + SD are shown.

Levels and specificities of pooled plasma, IgG1-3 and IgG4 fractions are shown in eFigure 1 (links.lww.com/NXI/A890). To confirm first that MuSK-IgG1-3 as well as IgG4 inhibited agrin-induced AChR clustering, myotubes were pre-exposed overnight to DMEM alone, agrin, or agrin plus 0.5 nM of the 2 MuSK-Ab preparations. AChR clusters were counted as previously described.^13^ Figure 1A shows that both IgG preparations inhibited AChR clustering, as shown previously. The effect of IgG1-3-Abs on MuSK phosphorylation had not been tested previously. For this, the myotubes were incubated for only 40 minutes before lysing the cells and immunoprecipitating MuSK. MuSK-IgG4 prevented MuSK phosphorylation induced by agrin, whereas surprisingly, phosphorylation was not reduced by IgG1-3 (Figure 1B). Moreover, IgG1-3, unlike IgG4, induced MuSK phosphorylation even in the absence of agrin.

One possibility was that IgG1-3–induced, agrin-independent, phosphorylation was not associated with phosphorylation of DOK7, the second molecule in the clustering pathway. In a parallel experiment, DOK7 phosphorylation was tested. As expected, MuSK-IgG4 inhibited agrin-induced DOK7 phosphorylation compared with agrin alone and had no effect in the absence of agrin. Conversely, phosphorylation of DOK7 was significantly increased (*p* < 0.001) by MuSK-IgG1-3 even in the absence of agrin (*p* < 0.05), suggesting a direct activation of MuSK and the downstream pathway by the antibodies (Figure 1C).

### Agrin and MuSK-IgG1-3 Induced a Similar Phosphorylation Time Course for MuSK, DOK7, and βAChR While Failing to Increase AChR Clustering

**Figure 2:**
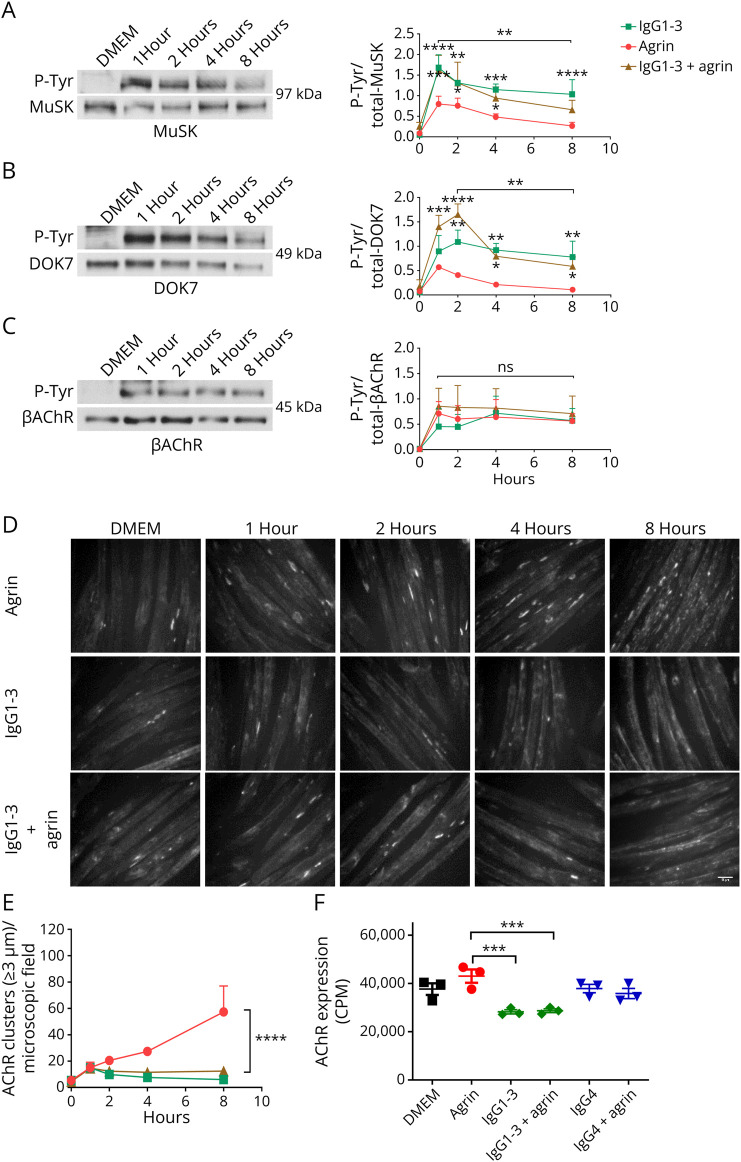
Time Course of Phosphorylation of MuSK, DOK7, and βAChR After Agrin or IgG1-3–Treated Myotubes Myotubes were exposed to agrin alone or MuSK-IgG1-3 with or without agrin, and incubation stopped at different time points. MuSK (A) and DOK7 (B) were immunoprecipitated as for Figure 1, and the βAChR (C) pulled-down by streptavidin-conjugated bungarotoxin. On the left, representative Western blots showing phosphotyrosine bands (top) and corresponding expression (bottom) of MuSK (97 kDa), DOK7 (49 kDa), and βAChR (45 kDa). On the right, pooled results of 2 experiments showing MuSK, DOK7, and βAChR phosphorylation time courses. (D) In parallel, there was increasing AChR clusters over time in agrin alone and reduced clusters in the presence of MuSK-IgG1-3. Scale bar = 50 μm. (E) Summary of the results. (F) Surface AChRs were measured with ^125^I-α-bungarotoxin after 16 hours incubation with the agrin and MuSK-Abs, showing modest reduction in the presence of IgG1-3 compared with DMEM, agrin or IgG4 MuSK-Abs. Two-way ANOVA for time was used. Mean + SEMs are shown.

To examine further, a time course was performed for MuSK, DOK7, and βAChR (representing the most phosphorylated AChR subunit). For MuSK and DOK7, phosphorylation increased sharply in the first 1–2 hours and then fell to around 50% at 8 hours (Figure 2, A and B). Phosphorylation of βAChR rose in the first hour and that level was maintained up to 8 hours (Figure 2C) in IgG1-3 with or without agrin. In parallel experiments, the number of AChR clusters progressively increased over time in the myotubes exposed to agrin, but there was no clustering in the presence of MuSK-IgG1-3 (Figure 2, D and E).

It was possible that the sustained phosphorylation of MuSK and DOK7 had an effect on the number of surface AChRs. After overnight incubation, myotubes were exposed to ^125^I-α-bungarotoxin to label the membrane AChRs. Compared with DMEM or agrin alone, there were reductions (25% and 33%; *p* = 0.001; Sidak multiple comparisons test) after incubation in IgG1-3 but not in IgG4 (Figure 2F).

### MuSK-IgG1-3 Impairs AChR Microcluster Formation and Fails to Sustain Their Full Maturation

**Figure 3:**
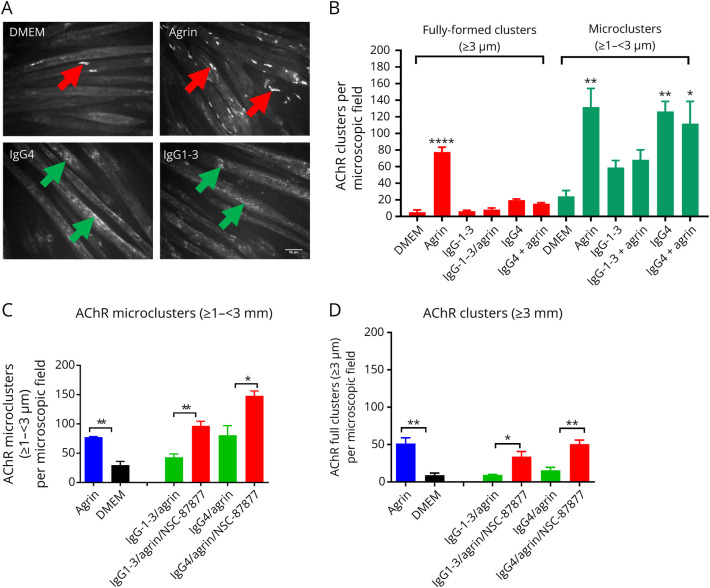
AChR Microclusters Are Reduced in IgG1-3–Treated Myotubes Myotubes were exposed to agrin, MuSK-IgG4, and IgG1-3 for 16 hours, and AChR clusters stained with Alexa Flour 594 α-bungarotoxin. Clusters were counted with ImageJ software using different thresholds for cluster length (≥1 – <3 µm for microclusters; ≥3 µm for fully formed clusters). (A) Representative images of AChR clusters. A few fully formed clusters form spontaneously on the myotube surface in DMEM or IgG4-Abs but, as expected, are increased substantially after agrin exposure (red arrows). By contrast, only AChR microclusters (green arrows) were present in myotubes treated with IgG1-3 or IgG4 MuSK-Abs. In this case, fully formed clusters were rarely detectable. (B) However, the results of 3 experiments showed that the AChR microclusters were reduced by approximately 50% in the presence of IgG1-3 compared with either agrin alone or IgG4-treated cells. Scale bar represents 50 µm. One-Way ANOVA with multiple comparisons against DMEM. (C) SHP2 inhibition by NSC-87877 increased the number of AChR microclusters to levels comparable or greater than agrin alone and (D) also the numbers of fully formed clusters. Two-sided t tests for comparisons at each IgG concentration. One-Way ANOVA with multiple comparisons against DMEM. Mean + SEMs are shown for all experiments.

The phosphorylation experiments did not throw light on how MuSK-IgG1-3 impaired clustering. We asked whether these antibodies could affect the formation and/or stability of the AChR clusters themselves, analyzing the numbers of smaller immature/developing microclusters.^14^ Few spontaneous AChR clusters were found in DMEM alone (<10 per field), but as expected, there were 70/field after agrin exposure (Figure 3A, agrin, red arrows). Microclusters were increased in agrin alone or with IgG4 but were reduced by 50% in IgG1-3, suggesting that there might be an underlying defect in AChR clustering, distinct from that of IgG4.

We previously showed that NSC-87877, a SHP2 inhibitor, restored AChR clusters in the presence of total IgG or IgG4 MuSK-Abs.^13^ Here, the effect of NSC-87877 was further tested on fully formed (Figure 3C) microclusters (Figure 3D). The drug effectively increased the numbers of clusters and microclusters in both IgG1-3 and IgG4, consistent with an effect that normalized AChR clustering.

### Transcriptional Modifications Induced by MuSK-Abs

To try to identify additional proteins/pathways that could be involved in the effects of IgG1-3, myotubes were exposed for 2.5 hours to DMEM, MuSK-IgG1-3, or total IgG from a healthy individual. Intracellular RNA was extracted, and gene expression in DMEM alone compared with MuSK-IgG1-3 or healthy IgG. A total of 102 gene transcripts were upregulated and 15 were downregulated at least 2 log-fold by MuSK-IgG1-3. After subtracting transcripts in healthy IgG from those for MuSK-IgG1-3, cytokines and transcriptional factors (e.g., IL6, TNF, Cxcl5, Fos, and Ras) were highly represented in the IgG1-3–treated myotubes (see eTable 1 (links.lww.com/NXI/A891) IgG1-3 v control). The only known gene in the AChR clustering pathway was Rho GTPase, upregulated 3.8 log-fold; but with an enzyme assay, agrin, IgG1-3, and IgG4 MuSK-Abs all increased Rho GTPase enzyme activity equally (data not shown).

As the effects of NSC-87877 might relate to the activation of noncanonical pathways, transcriptomic analysis was also performed on myotubes exposed to the drug. The cytochrome 450 family (Cyp1a1 and Cyp1b1), the Ahrr repressor, and Sic39a10 Zinc transporter were upregulated >2 log-fold by NSC-87877, suggesting an induction of metabolic/detoxicating pathways. Of possible relevance to the clustering of the AChRs, however, was neuregulin-1 (Nrg1), but this was just below threshold.

## Discussion

It is widely accepted that the IgG4 MuSK-Abs form the highest proportion of MuSK-Abs in patients with MG, inhibit agrin-induced MuSK phosphorylation (and DOK7 phosphorylation as shown here), and, consequently, the phosphorylation and clustering of the AChRs. The potential pathogenic pathway of the smaller population of MuSK-IgG1-3, however, is unclear. Here, we confirm that IgG1-3-Abs also inhibit AChR clustering, but their effects differ from those of IgG4, may involve a different pathway, and their effect can be counteracted by SHP2 inhibition. Overall, the results suggest that there is more to learn about the pathways involved in both AChR cluster formation and the pathophysiology of MuSK-MG.

As known from previous studies, MuSK-IgG4 antibodies undergo Fab-arm exchange in vivo becoming functionally monovalent, and their Fab fragments inhibit agrin-induced MuSK phosphorylation and AChR clustering.^9,10^ Recent studies have used engineered monovalent or divalent monoclonal antibodies, derived from MuSK-MG patient B cells, to explore the roles of subclass and valency. These found that passive transfer of monovalent MuSK-Abs rapidly induced clinical and electromyographical defects in mice, whereas only 1 of the 2 divalent antibodies produced the same phenotype and more gradually. Similarly, monovalent MuSK-Abs inhibited MuSK phosphorylation (maximal at 7.7 nM), whereas the same antibodies in divalent form increased MuSK phosphorylation and a partial increase rather than an inhibition of AChR clustering.^15-17^ We did not find evidence of AChR clusters in the MuSK-IgG1-3–treated myotubes but did find substantial inhibition of clustering. The differences may relate to the use of lower concentrations (0.5 nM MuSKAb activity) of native IgG1-3, rather than cloned antibodies.

MuSK-Abs seemed to impair the transition from unclustered AChRs to microclusters and then to fully matured clusters, but this could be due to a mechanism acting independently of the canonical clustering pathway.^6,7^ There are some parallels between the results reported here and those found with a mutation in the tyrosine kinase domain of MuSK, A617V.^18^ In cells constitutively expressing MuSK A617V, MuSK phosphorylation was increased, whereas AChR clusters were reduced, but in this case, microclusters were increased over the numbers in medium alone. It was concluded that overactive MuSK phosphorylation impairs the formation of large clusters/true NMJ morphology, similar to findings here.

We showed earlier that NSC-87877 alone induces MuSK phosphorylation, enhances the effect of agrin, and overcomes the inhibitory effect of MuSK-IgG4.^13^ Here, we show that NSC-87877 also overcomes the inhibitory effects of IgG1-3. The drug may circumvent the effects of IgG1-3, acting through separate pathways that regulate the formation and maturation of AChR clusters. NSC-87877 was also shown to enhance AChR stabililty,^19^ but whether it has any effect on MuSK membrane expression is not clear.

We hoped that changes in RNA transcripts might shed light on how IgG1-3 or NSC-87877 worked, but the results were disappointing. Many of the most upregulated substances were inflammatory mediators, and although some have been found to act at the neuromuscular junction (e.g., CXCl12 interacting with CXCR4),^20^ these were not identified in the current analysis. RNA for Rho GTPase was upregulated by MuSK-IgG1-3, but the enzyme activity was not different between agrin, IgG1-3, and IgG4 antibody-treated myotubes (data not shown). Three main pathways are now known to be involved in the formation and maintenance of the neuromuscular junction (reviewed in references 7, 21). Wnt signaling^22-25^ and BMP4 modulate the transcription of genes involved in AChR clustering^26^ including MUSK, DOK7, and Wnt11, but none of these were changed in MuSK or NCS-87877-treated cells. NSC-87877 did marginally upregulate neuregulin-1, which is involved in full cluster maturation,^27,28^ but whether any of these pathways affect the formation of microclusters is not known. Overall, the results of the transcriptomic analysis did not allow the development of a new hypothesis of the mechanisms by which MuSK-IgG1-3-Abs affect AChR clusters or how NSC-87877 protects them.

In vivo, divalent IgG1-3 MuSK-Abs might activate complement-mediated damage or induce internalization of MuSK. MuSK serum antibodies (both IgG1-3 and IgG4) activated complement on MuSK-expressing HEK cells,^29^ and this possibility needs to be explored in in vivo models. Divalent binding of MuSK by IgG1-3-Abs, leading to internalization by endocytosis and proteolysis, is conceptually likely (and has been demonstrated with other IgG1-3 antibodies, e.g., reference 30), but careful studies using MuSK-transfected HEK cells failed to find any internalization by either IgG1-3 or IgG4 MuSK-Abs or their Fab fragments, unless the Fab fragments were divalently linked by anti-human IgG antibodies.^10^ Nevertheless, we tried to test MuSK-IgG1-3 on the C2C12 myotubes, permeabilizing the cells with acetone to detect MuSK-IgG internally, but MuSK expression is low on the cells, and it was not possible to provide a conclusive answer (Cao et al. unpublished). In this context, it is interesting that exposure to agrin alone was shown to cause internalization of MuSK^31,32^; the reduced phosphorylation over time in the presence of agrin alone (Figure 2) would be consistent with this, but in the presence of IgG1-3 MuSK antibodies, the signal was largely retained suggesting little change in MuSK expression.

There are several limitations of this study. We did not examine the MuSK epitopes of the serum MuSK-Abs that we studied, and it is possible that they differ from those of IgG4. The control IgG for the transcriptomic analysis was not IgG4-depleted, and this might affect the results. Cloned human MuSK antibodies would have been very valuable, particularly for the transcriptomic analyses, although one has to consider that patient antibodies are polyclonal, binding to various epitopes, and monoclonal antibodies may not be representative of the predominant serum antibody.^16^ Despite these limitations, the results should stimulate further research not only on MuSK-MG but also on the additional pathways involved in the physiology of the neuromuscular junction.

## Glossary

AChR-Abs: acetylcholine receptor antibodies
AChRs: acetylcholine receptors
DOK7: Downstream of Kinase 7
LRP4: lipoprotein receptor-related protein 4
MG: myasthenia gravis
MuSK: muscle-specific kinase
NMJ: neuromuscular junction
